# Novel putative drivers revealed by targeted exome sequencing of advanced solid tumors

**DOI:** 10.1371/journal.pone.0194790

**Published:** 2018-03-23

**Authors:** Antonio Pannuti, Aleksandra Filipovic, Chindo Hicks, Elliot Lefkowitz, Travis Ptacek, Justin Stebbing, Lucio Miele

**Affiliations:** 1 Stanley S. Scott Cancer Center, Louisiana State University Health Sciences Center, New Orleans, Louisiana, United States of America; 2 Department of Oncology, Imperial College of Medicine, London, United Kingdom; 3 Department of Genetics, Louisiana State University School of Medicine, New Orleans, Louisiana, United States of America; 4 Biomedical Informatics Key Component, Louisiana Clinical and Translational Sciences Center, Baton Rouge, Louisiana, United States of America; 5 Department of Microbiology, University of Alabama at Birmingham School of Medicine, Birmingham, Alabama, United States of America; 6 Informatics Institute, Center for Clinical and Translational Sciences, University of Alabama at Birmingham School of Medicine, Birmingham, Alabama, United States of America; University of Nebraska Medical Center, UNITED STATES

## Abstract

Next generation sequencing (NGS) is becoming increasingly integrated into oncological practice and clinical research. NGS methods have also provided evidence for clonal evolution of cancers during disease progression and treatment. The number of variants associated with response to specific therapeutic agents keeps increasing. However, the identification of novel driver mutations as opposed to passenger (phenotypically silent or clinically irrelevant) mutations remains a major challenge. We conducted targeted exome sequencing of advanced solid tumors from 44 pre-treated patients with solid tumors including breast, colorectal and lung carcinomas, neuroendocrine tumors, sarcomas and others. We catalogued established driver mutations and putative new drivers as predicted by two distinct algorithms. The established drivers we detected were consistent with published observations. However, we also detected a significant number of mutations with driver potential never described before in each tumor type we studied. These putative drivers belong to key cell fate regulatory networks, including potentially druggable pathways. Should our observations be confirmed, they would support the hypothesis that new driver mutations are selected by treatment in clinically aggressive tumors, and indicate a need for longitudinal genomic testing of solid tumors to inform second line cancer treatment.

## Introduction

Next generation sequencing (NGS) is rapidly becoming integrated into oncological practice and clinical research [1]. Targeted exome panels, whole exome sequencing and whole genome sequencing of tumor samples, often paired with germline DNA sequencing to distinguish somatic from germline mutations, have revealed great heterogeneity in the mutational landscape of human cancers [2–6]. NGS methods have also provided evidence for clonal evolution of cancers, with selection of new genetic variants during disease progression and treatment [7]. Use of NGS information for treatment planning and clinical trial enrollment is increasing but remains in its infancy. Longitudinal testing of mutational landscapes and gene expression profiles will be essential to the development of adaptive precision medicine in cancer.

While the number of variants associated with response to specific therapeutic agents keeps increasing, a major challenge in the field remains the identification of driver mutations as opposed to passenger (phenotypically silent or clinically irrelevant) mutations. Potentially actionable mutations include indels, copy number variants (CNVs) and single nucleotide variants (SNV). The phenotypic consequences of CNVs and indels are generally easier to predict than those of SNV. Predictive methods for the pathogenicity of SNV that fall within protein coding regions have been developed that use a variety of algorithms [8, 9]. Evolutionary conservation of amino acid residues [10] and protein structural prediction can be used to predict the consequences of SNV in protein coding regions [11], while data from the ENCODE project [12] can be used to predict the consequences of SNV in non-coding DNA regions [13]. Limitations of these approaches include tumor heterogeneity with the possible presence of rare subclones that fall below the limit of detection and the difficulty in predicting the functional interactions between different mutations existing in the same tumor. Despite these limitations, NGS methods are revolutionizing our understanding of cancer biology and cancer therapeutics, and rapid accumulation of data will improve our ability to link genotypes and phenotypes.

The average number of drivers in human tumors has been estimated to be in the range of 2 to 8 [14]. By combining coventional epidemiological studies with genome-wide sequencing data, Tomasetti et al. have recently shown that in lung and colorectal carcinomas only three driver gene mutations are likely be required for cancer development [15]. However, it remains unclear how often new drivers are selected over the course of therapy in different tumor types. These treatment-selected drivers may be responsible for treatment failure and/or disease recurrence [7]. If selection of new drivers is a common occurrence, re-biopsy and longitudinal genomic testing or circulating tumor DNA [16–20] would have to become integral part of adaptive cancer treatment.

To explore this question, we conducted targeted exome sequencing of advanced solid tumors from 44 patients with solid tumors including breast, colorectal and lung carcinomas, neuroendocrine tumors, sarcomas and others. We catalogued established driver mutations and putative new drivers as predicted by two distinct algorithms. The established drivers we detected were consistent with published observations. However, we also detected a significant number of mutations with driver potential never described before in each tumor type we studied. These putative drivers belong to key cell fate regulatory networks, including potentially druggable pathways. Should these observations be confirmed, they would support the hypothesis that new driver mutations are selected by treatment in clinically aggressive tumors, and indicate a need for longitudinal genomic testing of solid tumors to inform second line cancer treatment.

## Materials and methods

### Biospecimens

Patients received standard of care treatment at the London Oncology Clinic, London UK. Genomic analyses of their tumors were performed as part of their routine care. All patient signed a requisition form provided by the testing facility, thereby consenting to release their tissue and allowing for NGS testing. De-identified biospecimens from 44 advanced solid tumors (29 metastatic lesions and 14 primary or local recurrence lesions) were analyzed. Table 1 shows the basic demographics and pathological diagnoses of the cases we studied. Every patient had received at least one first-line standard of care chemotherapy regimen appropriate for their malignancies without experiencing a complete response. The majority of patients were heavily pre-treated with first and second-line chemotherapy.

**Table 1:** List of cases by pathological diagnosis and patient gender.

Tumor type	Number	Female	Male	Primary lesion	Metastatic lesion
Breast (Triple-negative)	11	11	0	2	9
Breast (Her2-enriched)	3	3	0	0	3
Breast (ER+)	2	2	0	1	1
Colorectal carcinoma	10	7	3	2	8
Lung Adenocarcinoma	2	2	0	1	1
Squamous carcinoma	2	2	0	1	1
Sarcomas	4	2	2	2	2
Neuroendocrine tumor	4	2	2	3	1
Cancer of unknown primary origin	2	2	0	0	1
Thymus adenocarcinoma	1	0	1	1	0
Gastric carcinoma	1	1	0	0	1
Chordoma	1	0	1	1	0
Krukenberg tumor (ovarian)	1	1	0	0	1
TOTAL	44	35	9	14	30

### Exome sequencing

We performed targeted exome sequencing using the SmartGen 421 NGS gene panel (gene list provided in S1 Table), by obtaining biopsies from the most recent sites of progressive disease (primary or metastatic lesion). 15 unstained slides from each case were submitted to an accredited (CLIA-certified) clinical sequencing vendor. Custom Haloplex^TM^ reagents were used to capture the regions of interest by hybridization to probes corresponding to target regions. Targets were then amplified to further enrich DNA libraries. Libraries were sequenced on an Ion Torrent Personal Genome Machine, and sequences were compared to the reference genome GRCh37/hg19. The coding regions and +/-5 base pairs of the introns of 421 genes were sequenced. Analytic sensitivity for SNP calls was 94.1%, with a 95% confidence interval of 69.2%-99.7%. Specificity was 100%. For introns and deletions sensitivity was 78.9%, with a 95% confidence interval of 62.2%-89.9%, and specificity was 99.994%. Copy number changes and chromosomal rearrangements were not detected by this test. The clinical bioinformatics pipeline was provided by Kew, Inc. (840 Memorial Drive, Cambridge, MA 02129, USA), as described by Eifert et al. [21]. Briefly, variant calling was performed via GENEKEEPER, a Kew proprietary tool. Nonsynonymous variants in canonical transcripts present in the UniProt database were deprioritized if they were present under certain conditions (e.g., at allele frequency >1%) in dbSNP, 1000 Genomes, ExAC databases, and then reprioritized using COSMIC. Variants were reported if present at a Mutant Allelic Fraction (MAF) ≥ 10%.

### Data analysis

All analyses were performed independent of any company. Variant lists obtained from the sequencing vendor (S2 Table) were redacted and sent to the Louisiana State University Health Sciences Center and University of Alabama at Birmingham collaborative team for analysis, retaining patient confidentiality. Variants were manually re-validated individually against HG-19 and then evaluated for a potential resulting cancer driver phenotype. Missense mutations were scored with two algorithms, CHASM and FATHMM, which are considered reliable predictors [22, 23]. Cancer-specific High-throughput Annotation of Somatic Mutations (CHASM) is a computational method based on a Random Forest classifier trained to discriminate between driver missense mutations, culled mainly from the curated COSMIC database, and *in silico* generated passenger missense mutations in genes found mutated at least once in large scale sequencing studies of different tumors [24]. The scores from the Random Forest classifier are used to generate Benjamini-Hochberg corrected p values, with the null hypothesis positing that the mutation being tested is not functionally related to tumor growth (passenger). The CHASM engine can be accessed at the CRAVAT web site (http://www.cravat.us).

The Functional Analysis through Hidden Markov Models (FATHMM) algorithm was first developed for the prediction of the functional effects of protein missense variants in inherited diseases [11]. This method relies on the fact that hidden Markov models (HMMs) can be used to capture position-specific information within a multiple sequence alignment of homologous sequences. Starting with a manually curated HMMs representing the alignment of conserved protein domain families, a weighted/species-specific method incorporating “pathogenicity weights” was devised. These weights were derived from the relative frequencies of disease-associated and functionally neutral amino acid substitutions mapping onto conserved protein domains. In a later adaptation, a cancer-specific weighting scheme was incorporated to potentiate the functional analysis of driver mutations, and the algorithm was also extended to evaluate mutations falling outside conserved protein domains [13, 25]. A web-based implementation of the cancer-specific model is available at http://fathmm.biocompute.org.uk.

High confidence potential drivers were defined as both having a ≤ -1.5 score in FAHTMM (corresponding to a specificity of 0.94 and a sensitivity of 0.80) and a p-value ≤ 0.03 (corresponding to a false discovery rate (FDR) ≤ 0.15) in CHASM. Low confidence potential drivers met only one of these requirements. Indels in tumor suppressor genes resulting in early termination of translation or a deleterious frameshift were considered high confidence potential cancer drivers. Mutations in acceptor and donor splice sites in tumor suppressor genes were considered high confidence drivers if 1) the substitution involved invariant nucleotides in splice consensus sequences and 2) the splice site was used for the generation of all the mature mRNAs from the particular gene. Considering that the driver potential of a particular variant is context-depended, the actual driver likelihood of mutated tumor suppressors is based on known cases of haploinsufficiency or on a “worst case scenario”, where in at least a fraction of the tumor cells events such as loss of heterozygosity or silencing of the wild type allele take place [26].

**Fig 1:**
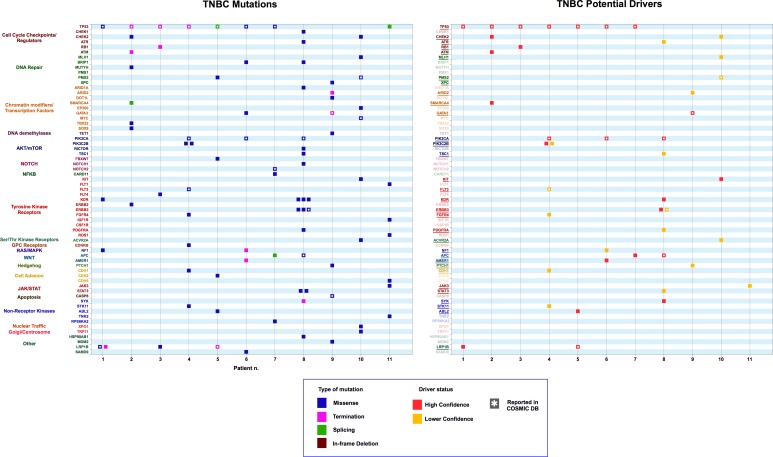
Left Panel: variants obtained from exome sequencing of tumor samples from Triple Negative Breast Cancer (TNBC) patients. Genes were grouped based on relevant biological activities/pathways. Right Panel: Variants identified as potential drivers (red: high-confidence drivers; gold: lower confidence drivers. Inset: Color coding scheme for types of mutations (mis-sense, termination, splice site, in-frame deletion), confidence of driver likelihood (high-confidence, lower confidence). Asterisks indicate mutations present in the COSMIC (Catalogue of Somatic Mutations in Cancer) database. The same coding scheme is used in Figs 2–5.

**Fig 2:**
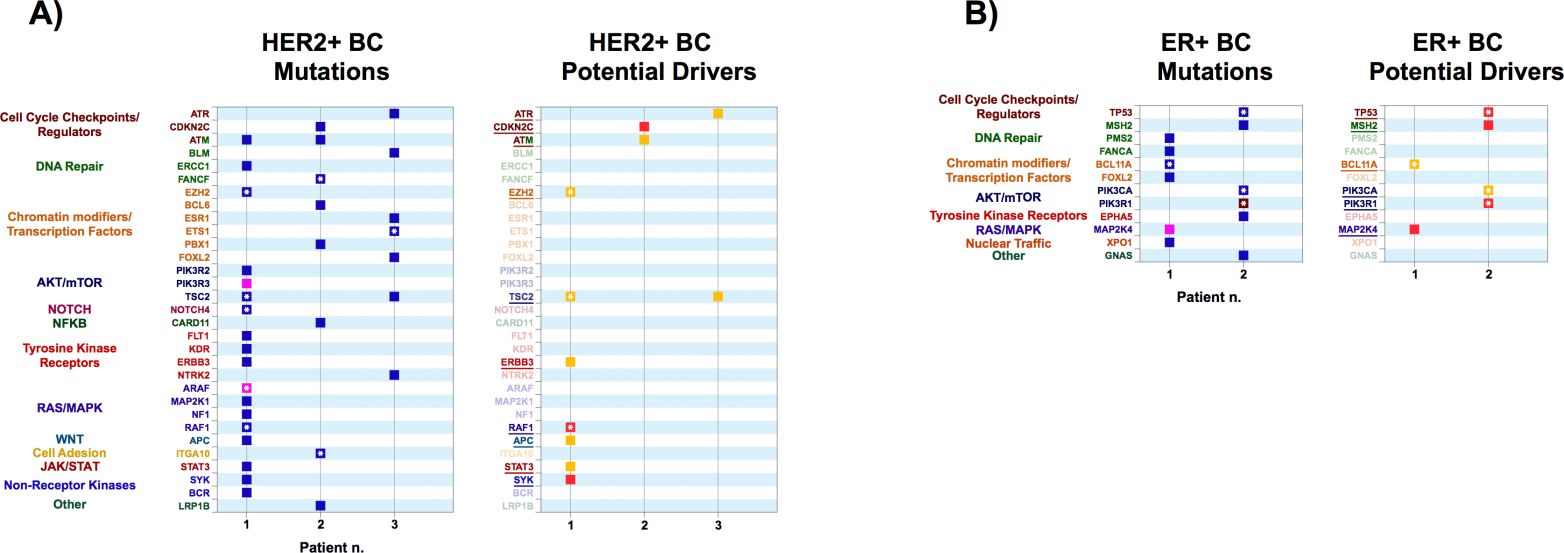
**A**) Left Panel: variants obtained from exome sequencing of tumor samples from Her2-enriched Breast Cancer (HER2+ BRC) patients. Right Panel: Variants identified as potential drivers. **B)** Left Panel: variants obtained from exome sequencing of tumor samples from ER positive Breast Cancer (ER+ BRC) patients. Right Panel: Variants identified as potential drivers. Color codes are as in Fig 1.

**Fig 3:**
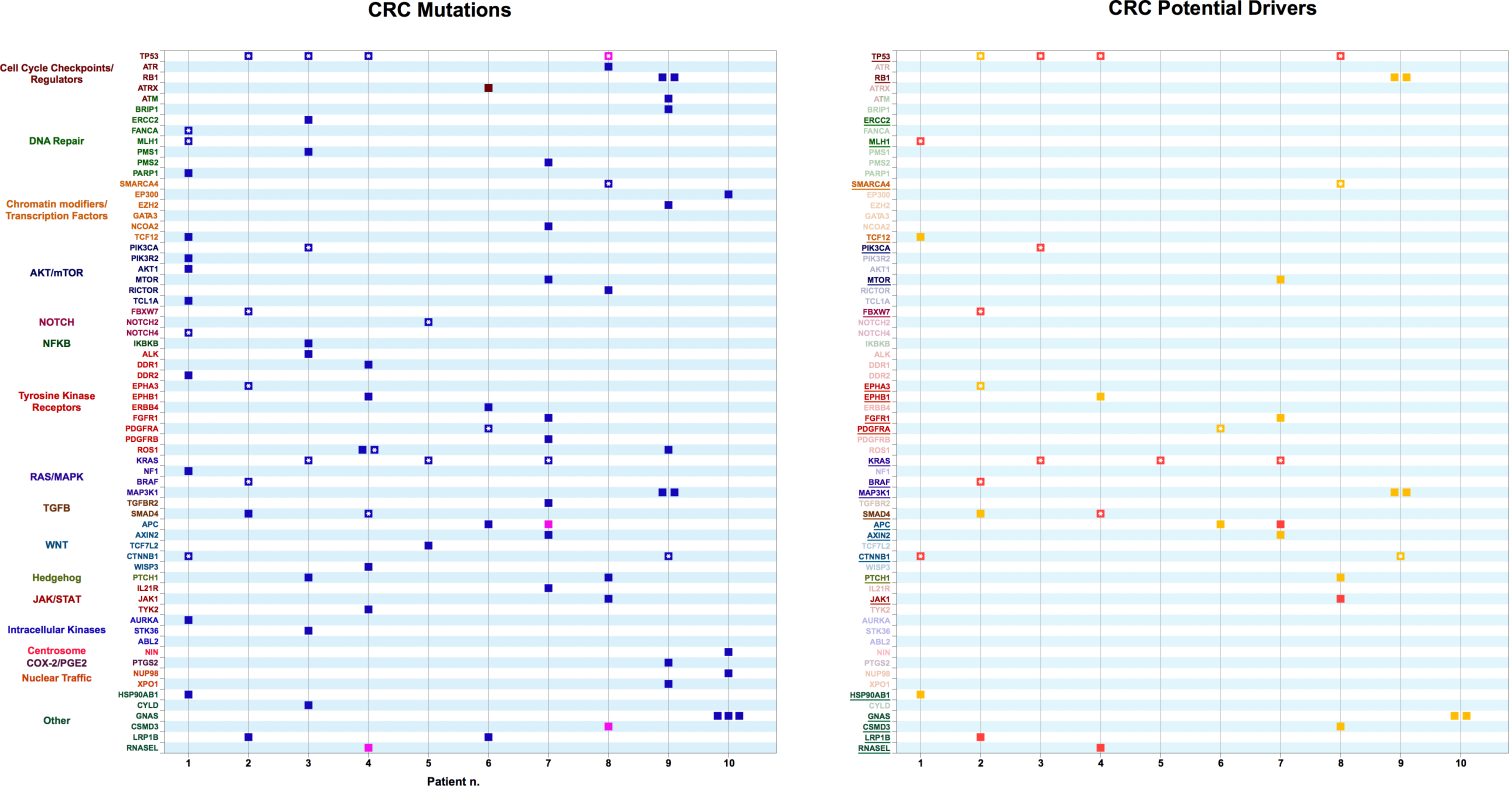
Left Panel: variants obtained from exome sequencing of tumor samples from Colorectal Cancer (CRC) patients. Right Panel: Variants identified as potential drivers. Color codes are as in Fig 1.

**Fig 4:**
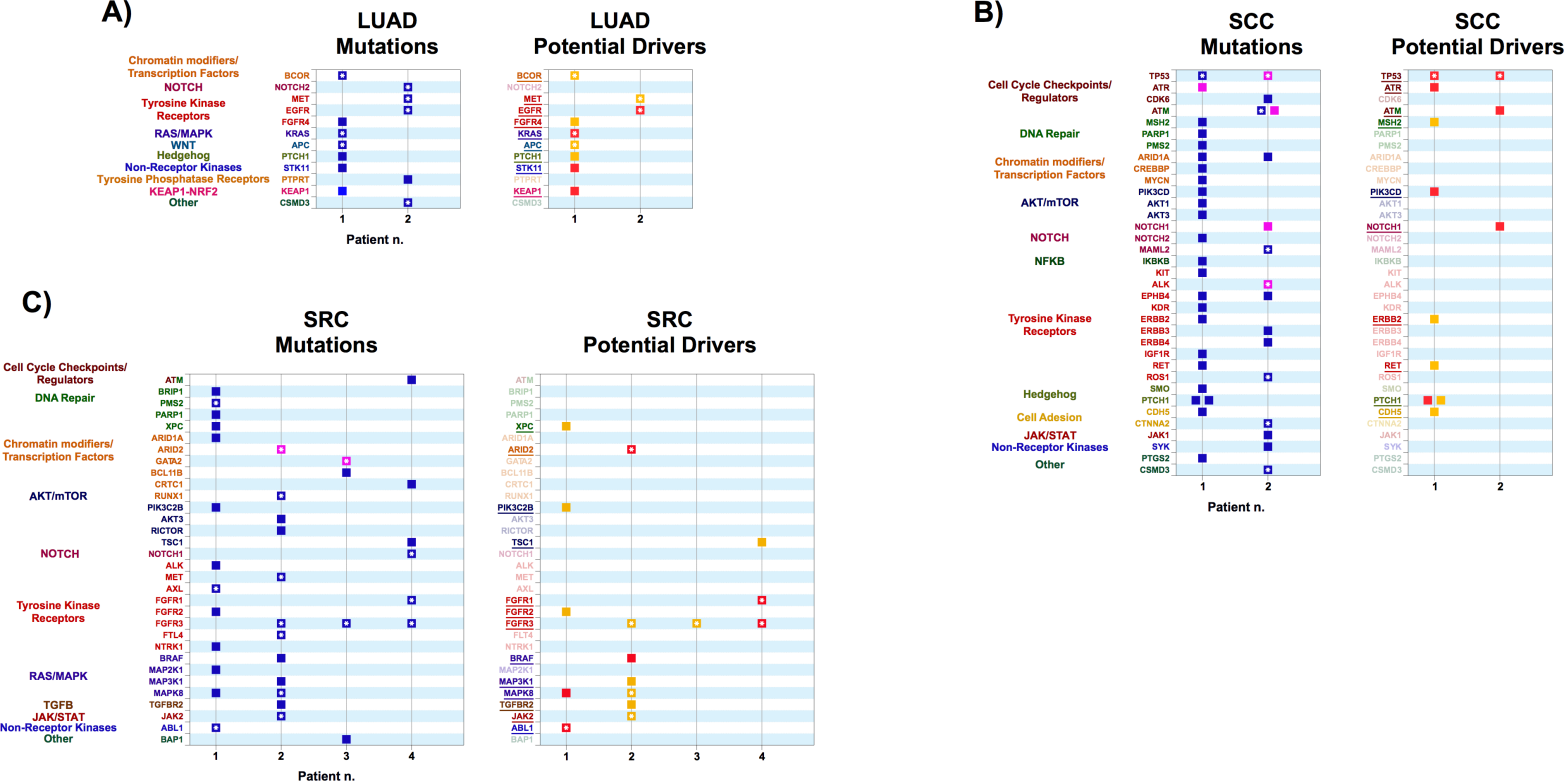
**A**) Left Panel: variants obtained from exome sequencing of tumor samples from Lung Adenocarcinoma (LUAD) patients, Right Panel: Variants identified as potential drivers. **B)** Left Panel: variants obtained from exome sequencing of tumor samples from Squamous Cell Carcinoma (SCC) patients. Right Panel: Variants identified as potential drivers. **C)** Left Panel: variants obtained from exome sequencing of tumor samples from Soft Tissue Sarcoma (SRC) patients. Right Panel: Variants identified as potential drivers. Color codes are as in Fig 1.

**Fig 5:**
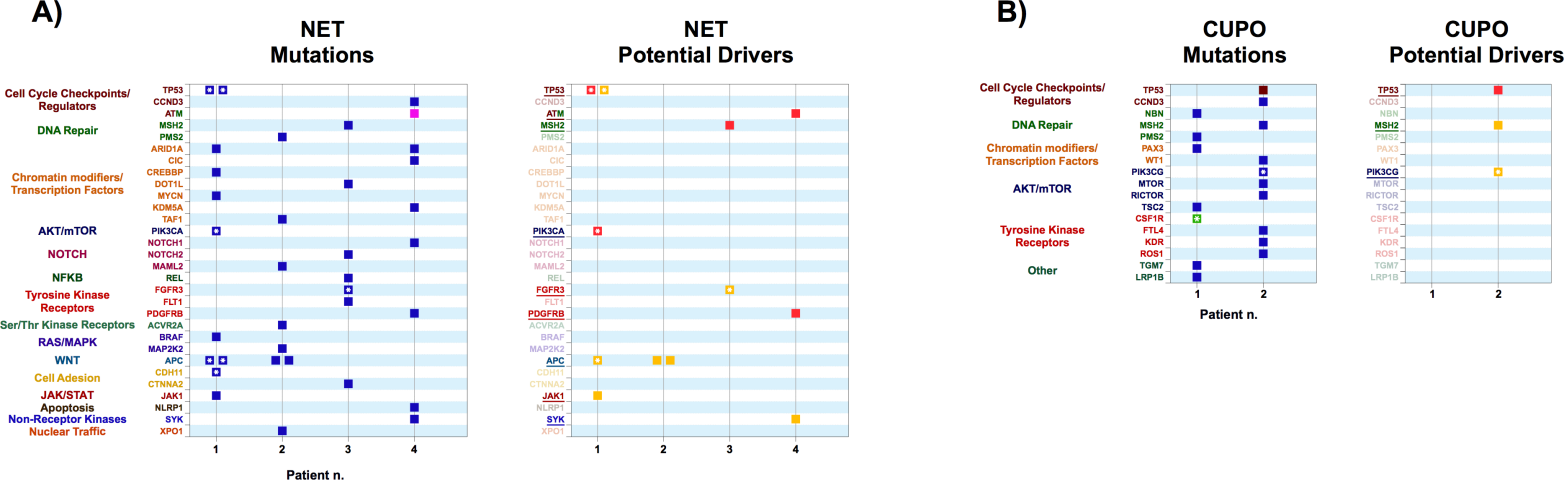
**A)** Left Panel: variants obtained from exome sequencing of tumor samples from Neuroendocrine Tumors (NET) patients. Right Panel: Variants identified as potential drivers. **B)** Left Panel: variants obtained from exome sequencing of tumor samples from patients with Carcinomas of Unknown Primary Origin (CUPO). Right Panel: Variants identified as potential drivers. Color codes are as in Fig 1.

Lastly, published reports regarding phenotypic consequences of particular mutations, included in S2 Table, were then used to inform the assessment and/or validate the scoring criteria. Mutations reported in the COSMIC database (http://cancer.sanger.ac.uk/cosmic), as of 12/2017 are indicated with asterisks in Figs 1–5.

We analyzed biospecimens from 44 deidentified, advanced solid tumors by targeted exome sequencing. A complete list of the variants identified in our study is presented in S2 Table. In our dataset, counts of potential driver mutations per patient (including known candidate drivers present in COSMIC and high-confidence putative drivers identified by both FATHMM and CHASM algorithms) form a distribution with a range of 0 to 9, a median of 3, and an average of 3.8. Evidence from well-studied cases indicates that a small number of drivers are sufficient to confer a neoplastic phenotype. For instance, only three driver mutations are likely required for the development of lung and colorectal cancers [15]. Our results suggest that the number of drivers in advanced tumors resistant to standard of care treatments may not necessarily be higher than in primary tumors. However, the number of novel high-confidence putative drivers identified in our samples supports the hypothesis that treatment selects resistant clones carrying new driver mutations [27–30]. Out of a total of 159 putative driver mutations, 41 (26%) were previously described in COSMIC and scored as high-confidence candidates by our analysis. Twenty (13%) were previously described in COSMIC and scored as low-confidence candidates. Forty-four mutations (28%) were previously undescribed and scored as high-confidence candidate drivers, while 58 mutations (36%) were previously undescribed and scored as low-confidence candidate drivers (See S2 Table).

The cancer subtypes represented in our study are discussed individually below.

#### Triple-negative breast cancer (TNBC)

TNBCs are a molecularly heterogeneous class of tumors [31] whose standard of care treatment is not yet based on molecular subtyping. In primary TNBC [32], TP53, PIK3CA, USH2A, MYO3A, PTEN and RB1 are among the most frequent genes with somatic exonic mutations, with TP53 also harboring splice site mutations that have significant impact on the mature transcripts. The 11 cases in our dataset showed considerable mutational heterogeneity, with the most common driver mutations occurring in the TP53 and PIK3CA genes (Fig 1A and 1B). All the mutations identified in these two genes were previously described in the COSMIC database. However, the case with the largest mutational load (19 mutations, Patient 8) carried no TP53 mutations or other putative driver mutations in DNA repair or cell cycle checkpoints. This case carried a known PIK3CA driver mutation plus a driver mutation in APC, E1309G, which falls in the mutation cluster region (MCR), where the majority of APC somatic mutations in familial adenomatous polyposis (FAP) and sporadic colorectal cancers occur [33]. Whether cases such as these may benefit from inhibition of the WNT and/or PIK3CA pathways deserves further investigation. Interestingly, the same case carried also 3 independent mutations in ERBB3, of which 2 scored as putative drivers. Whether such cases may be sensitive to Pertuzumab or other inhibitors of ErbB family tyrosine kinases remains to be determined. The presence of different variants in the same gene in samples 4 and 8 suggests possible intra-tumoral clonal heterogeneity. Samples 4 and 6 presented a H1047R substitution in the PIK3CA gene product, which is considered a strong predictor of tumor response to PI3K/AKT/mTOR inhibitors in heavily pretreated patients with advanced cancer [34]. Sample 4 also contained two different, previously undescribed mutations in PIK3C2B, a gene encoding the catalytic subunit of a PI3K isoform (type 2 beta). One of these mutations was a high-confidence putative driver, while the other was a low-confidence putative driver. Interestingly, it has been reported that 15% of mutations in genes in the PI3K-AKT-mTOR signaling axis across all tumor types are subclonal [27].

In our dataset, previously undescribed, high-confidence putative drivers were identified in CHEK2, RB1, ATM, SMARC4, KIT, ERBB3, KDR, APC, AMER1, PTCH1, CDH1, JAK3, SYK, ABL2 and LRP1 (Fig 1). Several of these novel putative drivers are in genes known to contain previously described drivers, including therapeutically relevant genes encoding protein kinases, Hedgehog pathway components and DNA repair components.

#### Her2-enriched breast cancer

We studied 3 metastatic lesions from Her2-enriched breast cancers (Fig 2A). Sample 1 was particularly rich in variants. It contained three putative driver mutations reported in the COSMIC database (in TSC2, EZH2 and RAF1), plus four previously undescribed low-confidence putative drivers in ERBB3, APC, STAT3 and SYK.

Sample 2 contained a novel high confidence putative driver in CDKN2C, which encodes p18, a CDK6 inhibitor, as well as an underscribed high confidence driver mutation in ATM. Palbociclib, a CDK4/6 inhibitor, is FDA-approved for the treatment of ER+/HER2- breast cancer and other CDK4/6 inhibitors are in development [35]. It is still unclear whether CDKN2C inactivating mutations sensitize tumors to CDK4/6 inhibitors. As for ATM, over 160 mutations have been described in COSMIC, both in hematological and solid malignancies. Several loss of function mutations in ATM are generally associated with shorter recurrence-free survival and chemotherapy resistance. However, they may increase sensitivity to platinum agents [36]. Sample 3 only had a single COSMIC-reported mutation in ETS1 which did not score as a putative driver, and 2 low-confidence putative drivers in ATR and TSC2, both previously undescribed. ATR loss of function has been reported to sensitize cancer cells to replication stress and DNA damage-induced G2/M cell cycle arrest [37].

#### ER+ breast cancer

The two ER+ breast cancers we studied contained 2 and 4 putative drivers respectively (Fig 2B). Sample 1 contained a previously reported mutation in BCL11A and a novel high-confidence putative driver in MAP2K4, the gene encoding c-Jun N-terminal kinase 1 (JNK1), which is a tumor suppressor in breast cancer as well as in other tumors [38, 39]. Sample 2 contained two previously described drivers in TP53 and PIK3R1, a negative regulator of PIK3CA, as well as a novel high-confidence putative driver in DNA repair gene MSH2 and a novel low-confidence putative driver in PIK3CA. PIK3R1 loss of function, as well as activating mutations in PIK3CA may be targeted with PIK3CA inhibitors or possibly inhibitors of kinases within the PI3K cascade, such as AKT or mTOR [40, 41].

#### Colorectal Cancer (CRC)

Most of the established or potential CRC driver mutations in our dataset have been reported in the literature (Fig 3). Recently, The Cancer Genome Atlas (TCGA) Project published a comprehensive molecular characterization of colon and rectal cancer [42]. The cases analyzed could be separated into two groups: hypermutated tumors, with mutation rates higher than 12 per 10^6^ bases and a majority of non-hypermutated cases, with a mutation rate less than 8.24 per 10^6^. Among the non-hypermutated tumours, the eight most frequently mutated genes were APC, TP53, KRAS, PIK3CA, FBXW7, SMAD4, TCF7L2 and NRAS. In the hypermutated tumours, ACVR2A, APC, TGFBR2, MSH3, MSH6, SLC9A9 and TCF7L2 were frequent targets of mutation, along with mostly BRAF(V600E) mutations. Three-quarters of hypermutated CRC had the expected high microsatellite instability, usually with hypermethylation and MLH1 silencing, and one-quarter had somatic mismatch-repair gene and DNA polymerase ɛ (POLE) mutations. Remarkably, TP53 and APC, two genes that were frequently mutated in the non-hypermutated cancers, were significantly less frequently mutated in hypermutated CRC [42]. Recently, CRC pathogenesis has been divided into at least two tumorigenic pathways, which produce molecularly distinct tumors [43, 44]. Hypermutated CRC tend to be associated with the non-traditional “serrated adenoma” pathway, which involves CpG island hypermethylation and microsatellite instability as well as BRAF mutations, which are uncommon in the traditional adenoma-carcinoma pathway [43, 44]. Such stratification of CRC patients is informative regarding therapeutical intervetions, since mismatch-repair status predicts clinical benefit of immune checkpoint blockade [45]. In our set, Sample 1 has the characteristics of a hypermutated class CRC [42], carrying a previously described MLH1 mutation, along with a previously described CTNNB1 mutation.

Novel high confidence putative drivers were identified in LRP1B (Patient 2), SMAD4 (Patient 4), RNASEL (Patient 4) APC (Patient 7) and JAK1 (Patient 8). The sample from Patient 2 carried a COSMIC-reported mutation in FBXW7, a gene encoding an F-box protein required for the degradation of multiple oncogenes including Notch1, Notch4, Myc and several others [46, 47]. This mutation scored as a high-confidence putative driver. Targeted exome sequencing is used to stratify advanced CRC patients for response to anti-EGFR mAbs therapy, which is much less effective in patients with tumors harboring activating mutations in KRAS, NRAS, BRAF, or PIK3CA and/or loss of function mutations in PTEN [48, 49], and FBXW7 [50]. In our dataset, patients 2, 3, 5 and 7 fall in this category. Clinical trials are under way to test combination therapies, such as anti-EGFR mAb necitumumab plus modified FOLFOX6 (oxaliplatin, folinic acid and 5-fluorouracil) [51] or treatments including BRAF inhibitors (plus anti-EGFR mAbs or/and other agents like irinotecan or MEK inhibitors) [52, 53] in advanced or metastatic CRC haboring KRAS or BRAF mutations, respectively. Patient 9 carries two distinct, previously undescribed mutations in both RB1 and MAP3K1. These four novel mutations all scored as low-confidence putative drivers, and may be evidence of clonal heterogeneity. Patient 10 is carrying three different mutations in GNAS, a complex locus encoding multiple transcripts including a G-protein α subunit [54] Two of the three scored as low-confidence putative drivers. Activating GNAS mutations are frequent in villous adenoma of the colorectum, while they are not commonly present in carcinomas. It has been suggested that these mutations play a transient role in carcinogenesis but are lost during tumor progression [55, 56].

#### Non-Small Cell Lung Cancer (NSCLC)

Non-Small-Cell lung cancer (NSCLC) includes two main histologic subtypes: adenocarcinomas (LUAD) and squamous cell carcinomas (LUSC). Most mutations in the two LUAD patients in our dataset are in genes commonly altered in this subtype (Fig 4A). Data from whole-exome sequencing of samples from 412 LUAD patients highlighted 18 statistically significant mutated genes: TP53, KRAS, KEAP1, STK11, EGFR, NF1, BRAF, SETD2, RBM10, MGA, MET, ARID1A, PIK3CA, SMARCA4, RB1, CDKN2A, U2AF1 and RIT1 [57]. Notably, mutations in KRAS (such as the one in Patient 1) were mutually exclusive with those in EGFR (Patient 2). EGFR is a well-known therapeutic target in LUAD and CRC. Patients with advanced lung adenocarcinomas harboring activating mutations in EGFR can benefit from the use of tyrosine kinase inhibitors (TKIs) [58]. Recent clinical trials have been focusing on patients with uncommon, poorly characterized mutations in EGFR and their responses to different TKIs [59]. In a post-hoc analysis of LUX-lung clinical trials, the TKI afatinib appeared to be effective in patients with the EGFR L861Q mutation (as in Patient 2 in our dataset) [60]. Unfortunately, development of resistance to TKIs is not uncommon, and can be determined by several possible molecular events [61]. Among them, a well-studied occurrence is an increase in the activity of membrane receptor MET, usually by amplification and enhanced expression. The LUAD in Patient 2 harbors a 504G>T mutation in MET, resulting in an E168D substitution, which is considered a moderately activating one [62]. Strategies for the combined targeting of EGFR and MET have been developed for such cases [61, 63].

The treatment of KRAS-mutant lung adenocarcinoma (as in Patient 1 in our dataset) is a major challenge. Since mutations in KRAS are mutually exclusive with those in EGFR, use of TKIs is not an effective option [64]. Targeting of the RAS pathway with MEK inhibitors, such as trametinib, has been evaluated, with poor results when the drug was used as single agent [65]. Coexisting mutations, such as loss of function in STK11 (also known as LKB1) and KEAP1 (as in Patient 1), can inform therapeutic decisions. In fact, mouse models of Kras-dependent NSCLC revealed that tumors with Kras and Lkb1 mutations, but not those with Kras and p53 mutations, showed selective response to the mitochondrial inhibitor phenformin as a single agent, resulting in prolonged survival [66]. The combined use of phenformin with the mTOR inhibitor MLN0128 has been proposed as a treatment strategy for NSCLC bearing concomitant mutations in the KRAS and STK11 genes [67]. Currently, efforts are underway to explore combination therapies in KRAS-driven NSCLC including MEK and FGFR1 inhibitors [68], or RAS pathway inhibitors and immunotherapy strategies [69].

#### Squamous Cell Carcinomas (SCC)

Our cases included two SCC cases, a primary and a metastatic lesion. Both carried known p53 mutations and were particularly rich in mutations (Fig 4B). The first case also carried a novel ATR mutation (see above, [37]), which scored as a high-confidence putative driver, and might be sensitive to DNA-damaging agents or inhibitor of DNA repair mechanisms. This sample harbored two distinct novel PTCH1 mutations, which scored as high and low confidence putative drivers respectively. PTCH1 is a major tumor suppressor in basal cell carcinoma [70–72] but allelic loss or loss of function mutations of PTCH1 are not unknown in squamous cell carcinomas [73].

The second case carried two distinct novel ATM mutations (see above [36]), one of which scored as a high confidence putative drivers, as well as a novel Notch1 mutation, which also scored as a high confidence putative driver.

#### Sarcomas (SRC)

Four soft tissue sarcomas were included in our dataset. Each carried at least one previously identified driver mutation (Fig 4C). Patient 1 carried an ABL1 mutation, while patient 2 carried a mutation in ARID2, a Swi/Snf component that participates in chromatin remodeling [74]. Of note, mutations in Swi/Snf components are present in approximately 20% of cancers, and this chromatin remodeling complex is considered an attractive therapeutic target [75]. Additionally, two cases carried novel high-confidence putative drivers. Patient 1 carried a mutation in MAPK8, the gene encoding c-Jun N-terminal kinase 1 (JNK1) [76]. Patient 2 carried a novel mutation in BRAF [77]. It is unclear what effect this mutation may have on sensitivity to RAF inhibitors such as vemurafenib. BRAF mutations have been described in follicular and dendritic cells sarcomas [78, 79].

#### Neuroendocrine tumors (NET)

Two of the 3 neuroendocrine tumors we studied carried previously described drivers in TP53, PIK3CA and APC in Patient 1 and FGFR3 in patient 3 (Fig 5B). The sample from patient 2 carried two distinct APC mutations, both scoring as low-confidence putative drivers. Interestingly, Patient 1 also carries two distinct APC mutations, both reported in COSMIC. In both cases, tumor clonal heterogeneity is a possible explanation. The possible role of the WNT-β-catenin pathway in such cases might be exploited through pharmacological inhibition of the wnt signaling [80]. Patient 3 carried a novel high-confidence putative driver mutation in MSH2, suggesting that inpaired DNA damage repair (DDR) may be a feature of this case. The single Small Cell Lung Carcinoma sample (SCLC, patient 4) we studied contained two novel high confidence putative driver mutations in ATM and PDGFRB (Fig 5A). Consistent with a defective DDR, this case contained 9 distinct mutations, all previously undescribed (Fig 5A).

#### Carcinomas of unknown primary origin (CUPO)

CUPOs make up approximately 3% of advanced solid tumors, and are generally treated empirically [81]. Gene expression profiling has been suggested as a tool to identify tissue of origin and guide treatment [81]. Exome sequence data on these tumors remain scant. The two CUPO cases in our dataset had distinct mutational profiles. The first carried mutations in PMS2, TSC2, CSF1R, LRP1B, NBN, PAX3 and TGM7. None of these scored as a putative driver, although the CSF1R mutation is reported in COSMIC. The second sample carried novel mutations in TP53, WT1, CCND3, MSH2, FLT4, RICTOR, ROS1, KDR and mTOR, as well as a COSMIC-reported PIK3CG mutation that scored as a low-confidence putative driver. The TP53 mutation scored as a high-confidence putative driver and the MSH2 mutation as a low-confidence putative driver (Fig 5C).

#### Miscellaneous cases

Single cases of thymic carcinoma cancer (TC), Krukenberg cell tumor (KRUK), gastric carcinoma (GC) and chordoma (CHORD) were included in our dataset. The TC carried KRAS, FLT1, PTEN, PTCH1, ATRX, DCC and TP53 mutations. The KRAS and TP53 were previously described in COSMIC and scored as high-confidence putative drivers. The PTEN, PTCH1 and ATRX mutations were novel, high-confidence putative drivers whilst the CSF1R mutation was a low-confidence putative driver. The GC carried no known mutations. It contained novel TP53, RNASEL and SMAD4 mutations that scored as high-confidence putative drivers, as well as ERBB2 and GRIN2A low-confidence putative drivers and an EDNRB mutation. ERBB2 (HER2/Neu) is overexpressed or amplified in a significant fraction of GC [82], particularly cancers with intestinal histology and cancers of the gastro-esophageal junction. In the ToGA trial [83], the addition of trastuzumab to chemotherapy significantly increased median overall survival compared to chemotherapy alone in HER2-positive GC. Whether activating mutations in ERBB2 can act as oncogenes in GC cases and whether they confer sensitivity to HER2 inhibitors requires further investigation. The CHOR carried PMS2, PARP1, CRTC1 and IL7R mutations. None of these scored as high- or low-confidence drivers, but the first two were described in COSMIC. The KRUK carried a CEBPA mutation described in COSMIC that scored as a high-confidence putative driver, as well as two novel high-confidence putative drivers in GATA1 and SMAD2, a low-confidence putative driver in ABL1, as well as KDM6A, EGFR, NOTCH2, GATA1, KAT6B, TNFAIP3, RPTOR, RUNX1 and TNFAIP3 mutations. The latter two were included in COSMIC.

## General considerations

Molecular profiling is increasingly used as a basis for treatment decisions. In a recent report, Carter et al. have shown that therapy recommendations based on molecular profiling can improve clinical outcomes, including overall survival [84]. Targeted exome sequencing is generating information heretofore unknown on the mutational landscapes of human malignancies. However, the usefulness of this data for clinical decision-making requires an understanding of the phenotypic consequences of novel sequence variants of unknown significance. Determining which variants may function as drivers without direct experimental evidence is difficult, and relies on the accuracy of predictive algorithms. As more data accumulate, the accuracy of predictive methods is bound to improve. Yet, the identification of driver mutations remains a difficult task [14, 15, 85].

**Fig 6:**
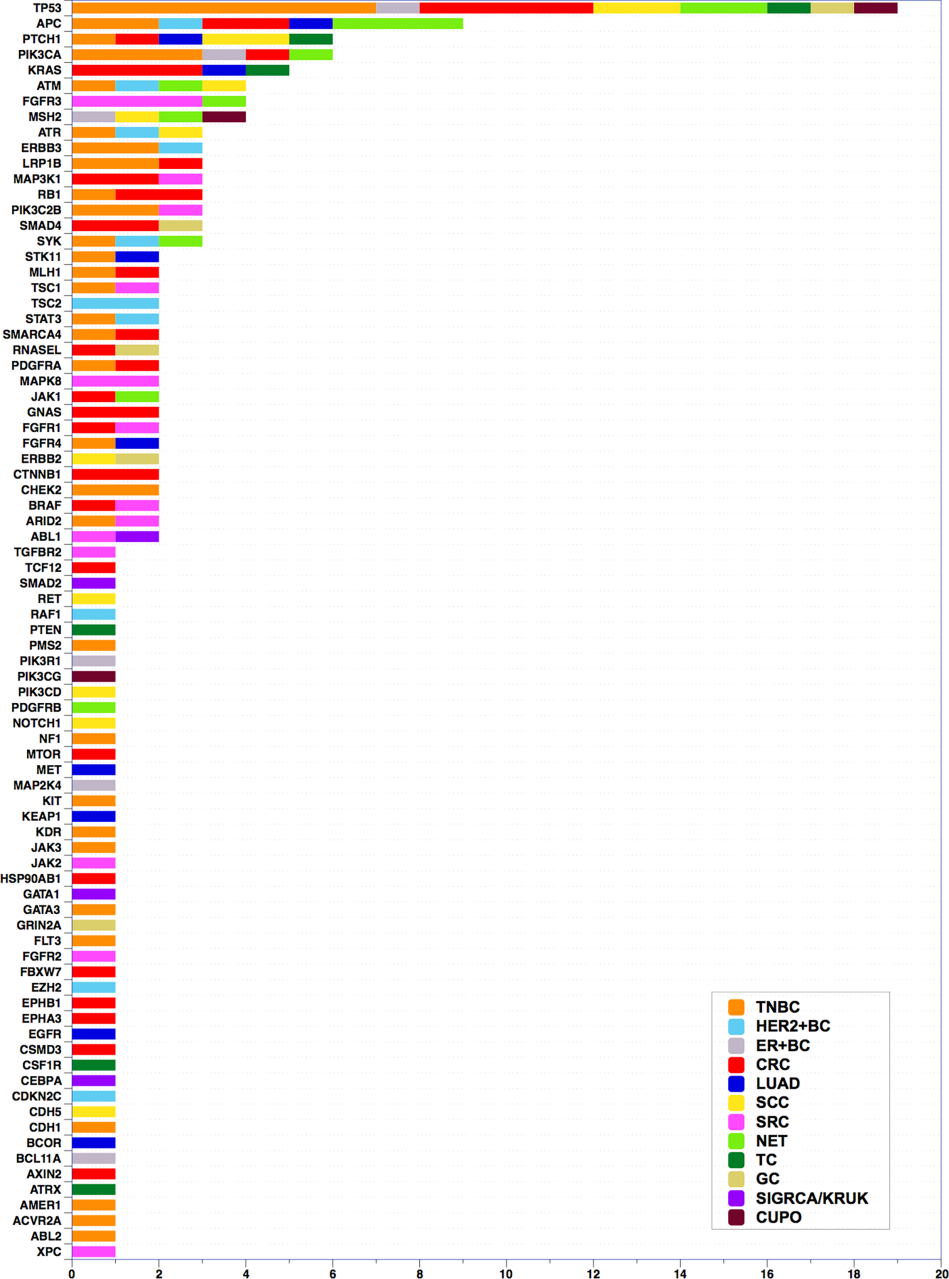
Absolute numbers of previously described and putative drivers identified in this study in different tumor types. Inset: color coding reflecting tumor types. Abbreviations: TNBC (Triple Negative Breast Cancer), HER2+BC (Her2-enriched Breast Cancers), ER+BC (ER-positive Breast Cancer), CRC (Colorectal Cancer), LUAD (Lung Adenocarcinoma), LUSC (Lung Squamous Cell Carcinoma), SCC (Squamous Cell Carcinoma), SRC (Soft Tissue Sarcoma), NET (Neuroendocrine Tumor), TC (Thymic Carcinoma), GC (Gastric Cancer), SIGRCA/KRUK (Signet Ring Adenocarcinoma/Krukenberg Cell Tumor), CUPO (Carcinoma of Unknown Primary Origin).

Fig 6 shows the distribution of known and putative drivers identified in our study among tumor types. Predictably, p53 mutations were the most commonly detected. Other DNA repair genes, such as ATM and MSH2, were also commonly mutated, as were PIK3CA and KRAS. Interestingly, the second most commonly mutated gene was APC, suggesting frequent involvement of the Wnt pathway in these advanced tumors. PTCH1 was mutated as frequently as PIK3CA, suggesting that the Hedgehog pathway may also be frequently involved in recurrent cancers.

**Fig 7:**
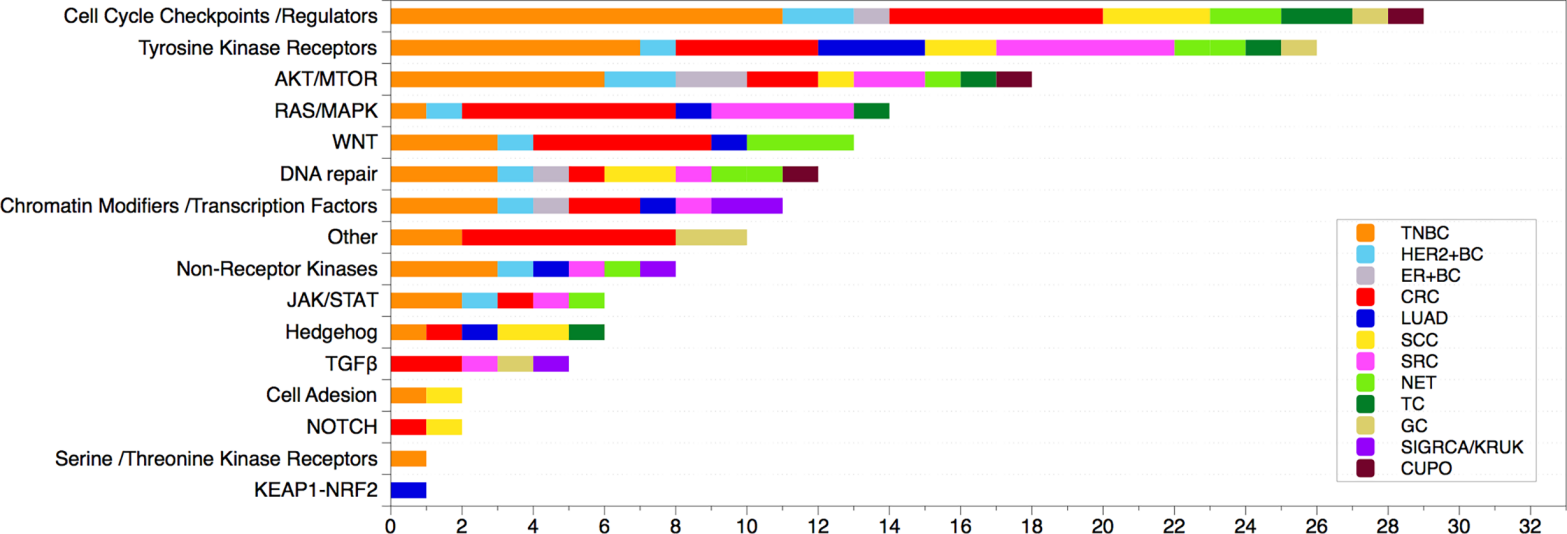
Previously described and putative drivers identified in this study grouped by functional classes or biological pathways. Pathway or functional class assignment was based on Gene Ontology supplemented by individual PathCards searches (http://pathcards.genecards.org/) for each gene. Inset: color coding reflecting tumor types. Abbreviations: TNBC (Triple Negative Breast Cancer), HER2+BC (Her2-enriched Breast Cancers), ER+BC (ER-positive Breast Cancer), CRC (Colorectal Cancer), LUAD (Lung Adenocarcinoma), LUSC (Lung Squamous Cell Carcinoma), SCC (Squamous Cell Carcinoma), SRC (Soft Tissue Sarcoma), NET (Neuroendocrine Tumor), TC (Thymic Carcinoma), GC (Gastric Cancer), SIGRCA/KRUK (Signet Ring Adenocarcinoma/Krukenberg Cell Tumor), CUPO (Carcinoma of Unknown Primary Origin).

The majority of known and putative driver mutations in our dataset can be classified as belonging to the following functional classes/pathways: Cell Cycle Checkpoints and Regulators, Tyrosine Kinase Receptors, AKT/mTOR, RAS/MAPK, WNT and DNA repair (Fig 7).

The patients in our study would fit a typical phase 1 oncology trial design exploring the safety of a new agent in “advanced solid tumors”. There appears to be very little tissue or organ-specificity to the pathways containing putative drivers. Conversely, even tumors of the same histologic subtype were genetically highly heterogeneous. This evidence supports the use of innovative clinical trial designs, such as “basket” trials in which patients are assigned to study arms based exclusively on genomic features or “umbrella” trials where patients are assigned to arms based on genomic features within a specific tumor subset. In several cases, multiple potential driver mutations in different pathways were present in the same sample. The phenotypic consequences of concomitant functional alteration of different pathways are difficult to predict in the absence of experimental data on crosstalk between these pathways and in the absence of gene expression profiling data. Several cases in our dataset contained 2 and even 3 different mutations in the same gene. Multiclonality is a possible explanation for such cases. Multiclonality may reduce the benefits of targeted therapies [86]. The phenotypic consequences of independent mutations in the same gene are difficult to predict, particularly in case of non-synonymous amino acid substitutions that don’t obviously disrupt protein structure. The overall mutational load showed remarkable variability between cases. Among the genes explored in this study, some tumors had as few as 3–5 mutations (e.g., TNBRC #1, CRC #5, NSCLC #2), while others had 18–24 or more (e.g., TNBRC #8, HER2 BRC #1, SCC #1). Mutational load itself can be clinically informative. Tumors with high mutational loads are thought to be good candidates for immunotherapy [45, 87, 88]. Often, these tumors contain one or more mutations in DNA repair and/or cell cycle checkpoint genes. Important questions that will need to be addressed in future, prospective studies include determining whether individual chemotherapy regimens or classes of drugs (e.g., platinum compounds, anthracyclines) are associated with the appearance of specific mutations, and whether radiation therapy, which in many cases is used prior to or concomitantly with chemotherapy, contributes to select specific driver mutations. The latter question would be particularly important in cases when radiation is used prior to chemotherapy. Ideally, in such cases mutational profiles and clonal composition ought to be determined after radiation therapy to dictate the choice of possible targeted therapies to use with chemotherapy or instead of it.

Limitations of our study include the following: 1) only a selected group of cancer-relevant, protein-coding genes were studied. Other protein-coding genes, noncoding RNA genes and other regulatory sequences were not included in this study. Mutations in regulatory sequences are increasingly recognized as relevant to cancer development and progression but are not routinely tested in clinical panels [89, 90]; 2) Copy number variations and transcript expression profiles were not part of the study; 3) We did not have access to information on relative frequencies of the variants identified, although variants were only reported if they were found in at least 10% of the reads and 4) Gene expression profiling by RNASeq or transcriptome-wide microarrays was not feasible.

Despite these limitations, our data confirm the remarkable molecular heterogeneity of histologically similar advanced tumors. The number of novel high-confidence putative drivers (26% of the total putative drivers in this study) and the number of cases carrying multiple mutations in the same genes suggests that advanced, treatment-resistant tumors select novel drivers without necessarily increasing the overall number of drivers, and that clonal heterogeneity is a common characteristics of these cases. Exome sequencing tests performed on primary surgical specimens may or may not capture mutations responsible for therapy resistance. Longitudinal sampling, whenever possible, or circulating tumor DNA may provide a data-driven strategy for adaptive planning of cancer therapy.

## Supporting information

S1 Table SmartGen™ whole exon 421 gene cancer panel. List of genes included in the targeted exome sequencing panel.
(DOCX)

S2 Table List of variants identified in 44 advanced solid tumors by targeted exome sequencing. Variants detected in genes listed in the SmartGen™ 421 NGS gene panel were evaluated for a potential resulting cancer driver phenotype as described in “materials and methods”. A continuous underline highlights high-confidence drivers, while a dashed underline identifies low confidence drivers. A shaded capital C (**C**) marks known mutations listed in the Cosmic database.
(DOCX)
