# Supplementary material for: Novel putative drivers revealed by targeted exome sequencing of advanced solid tumors
Source: PLoS One. 2018 Mar 23;13(3):e0194790. doi: 10.1371/journal.pone.0194790 (PMC5865730; doi:10.1371/journal.pone.0194790)
Supplement: S1 Table — List of genes included in the targeted exome sequencing panel. (DOCX) [file pone.0194790.s001.docx]

| **S1 Table.** SmartGen™ Whole Exon 421 Gene Cancer Panel. | | | |
| --- | --- | --- | --- |
|  |  |  |  |
|  |  |  |  |
| ABL1 | BCR | CRKL | ERCC5 |
| ABL2 | BIRC2 | CRLF2 | ERG |
| ACVR1B | BIRC3 | CRTC1 | ESR1 |
| ACVR2A | BLM | CSF1R | ETS1 |
| AFF1 | BLNK | CSMD3 | ETV1 |
| AKT1 | BMPR1A | CTCF | ETV4 |
| AKT2 | BRAF | CTNNA1 | EXT1 |
| AKT3 | BRD3 | CTNNA2 | EXT2 |
| ALK | BTK | CTNNB1 | EZH2 |
| AMER1 | BUB1B | CYLD | FAM46C |
| APC | C11orf30 | CYP2C19 | FANCA |
| AR | CARD11 | CYP2D6 | FANCC |
| ARAF | CASP8 | DAXX | FANCD2 |
| ARFRP1 | CBFB | DCC | FANCE |
| ARID1A | CBL | DDB2 | FANCF |
| ARID2 | CCND1 | DDIT3 | FANCG |
| ARNT | CCND2 | DDR1 | FANCL |
| ASCL4 | CCND3 | DDR2 | FAS |
| ASXL1 | CCNE1 | DICER1 | FBXW7 |
| ATM | CD79A | DNMT3A | FGF10 |
| ATR | CD79B | DOT1L | FGF14 |
| ATRX | CDC73 | DST | FGF19 |
| AURKA | CDH1 | EDNRB | FGF23 |
| AURKB | CDH2 | EGFR | FGF3 |
| AURKC | CDH5 | EML4 | FGF4 |
| AXIN2 | CDH11 | EP300 | FGF6 |
| AXL | CDK4 | EPHA3 | FGFR1 |
| BAP1 | CDK6 | EPHA5 | FGFR2 |
| BARD1 | CDK8 | EPHA7 | FGFR3 |
| BCL10 | CDK12 | EPHB1 | FGFR4 |
| BCL11A | CDKN1B | EPHB4 | FH |
| BCL11B | CDKN2A | EPHB6 | FLCN |
| BCL2 | CDKN2B | ERBB2 | FLI1 |
| BCL2L1 | CDKN2C | ERBB3 | FLT1 |
| BCL2L2 | CEBPA | ERBB4 | FLT3 |
| BCL6 | CHEK1 | ERCC1 | FLT4 |
| BCL9 | CHEK2 | ERCC2 | FN1 |
| BCOR | CIC | ERCC3 | FOXA1 |
| BCORL1 | CREBBP | ERCC4 | FOXL2 |
|  |  |  |  |
|  |  |  |  |
|  |  |  |  |
|  |  |  |  |
| FOXO1 | INHBA | MCL1 | NKX2-1 |
| FOXP1 | IRF4 | MDM2 | NLRP1 |
| G6PD | IRS2 | MDM4 | NOTCH1 |
| GATA1 | ITGA10 | MED12 | NOTCH2 |
| GATA2 | ITGB2 | MEF2B | NOTCH4 |
| GATA3 | ITGB3 | MEN1 | NPM1 |
| GDNF | JAK1 | MET | NRAS |
| GID4 | JAK2 | MITF | NTRK1 |
| GNA11 | JAK3 | MLH1 | NTRK2 |
| GNA13 | JUN | MLL | NTRK3 |
| GNAQ | KAT6A | MLL2 | NUMA1 |
| GNAS | KAT6B | MMP2 | NUP214 |
| GPC6 | KDM5A | MN1 | NUP93 |
| GPR124 | KDM5C | MPL | NUP98 |
| GRIK3 | KDM6A | MRE11A | PAK3 |
| GRIN2A | KDR | MSH2 | PALB2 |
| GSK3B | KEAP1 | MSH6 | PARP1 |
| HCAR1 | KIAA1804 | MST1R | PAX3 |
| HCN1 | KIT | MTOR | PAX5 |
| HGF | KLF5 | MTR | PAX7 |
| HIF1A | KLF6 | MTRR | PAX8 |
| HLF | KLHL6 | MUC16 | PBRM1 |
| HNF1A | KRAS | MUTYH | PBX1 |
| HNF4A | LAMP1 | MYC | PDGFRA |
| HRAS | LCK | MYCL1 | PDGFRB |
| HSP90AA1 | LPP | MYCN | PDK1 |
| HSP90AB1 | LRP1B | MYD88 | PGAP3 |
| ICK | LTF | MYH9 | PHOX2B |
| IDH1 | MAGEA1 | MYH11 | PIK3C2B |
| IDH2 | MAGI1 | NBN | PIK3CA |
| IGF1R | MALT1 | NCOA1 | PIK3CB |
| IGF2 | MAML2 | NCOA2 | PIK3CD |
| IGF2R | MAP2K1 | NCOA4 | PIK3CG |
| IKBKB | MAP2K2 | NF1 | PIK3R1 |
| IKBKE | MAP2K4 | NF2 | PIK3R2 |
| IKZF1 | MAP3K1 | NFE2L2 | PIK3R3 |
| IL2 | MAP3K7 | NFKB1 | PIM1 |
| IL21R | MAPK1 | NFKB2 | PKHD1 |
| IL6ST | MAPK8 | NFKBIA | PLAG1 |
| IL7R | MBD1 | NIN | PLCG1 |
|  |  |  |  |
|  |  |  |  |
|  |  |  |  |
| PML | SETD2 | TNFAIP3 |  |
| PMS1 | SF3B1 | TNFRSF14 |  |
| PMS2 | SGK1 | TNK2 |  |
| POT1 | SH2D1A | TOP1 |  |
| POU5F1 | SLIT2 | TP53 |  |
| PPARG | SMAD2 | TP63 |  |
| PPP2R1A | SMAD4 | TPR |  |
| PRDM1 | SMARCA4 | TRIM33 |  |
| PRKAR1A | SMARCB1 | TRIP11 |  |
| PRKDC | SMO | TRRAP |  |
| PSIP1 | SOCS1 | TSC1 |  |
| PTCH1 | SOX2 | TSC2 |  |
| PTEN | SOX9 | TSHR |  |
| PTGS2 | SOX10 | TYK2 |  |
| PTPN11 | SOX11 | UBR5 |  |
| PTPRD | SPEN | USP9X |  |
| PTPRT | SPOP | VHL |  |
| RAF1 | SRC | WAS |  |
| RALGD5 | STAG2 | WBSCR17 |  |
| RARA | STAT3 | WISP3 |  |
| RASA1 | STAT4 | WT1 |  |
| RB1 | STK11 | XPC |  |
| REL | STK36 | XPO1 |  |
| RET | SUFU | ZNF384 |  |
| RHOH | SYK | ZNF521 |  |
| RICTOR | TAF1 |  |  |
| RNASEL | TAF1L |  |  |
| RNF2 | TAL1 |  |  |
| RNF43 | TBX22 |  |  |
| ROS1 | TCF12 |  |  |
| RPS6KA2 | TCF3 |  |  |
| RPTOR | TCF7L1 |  |  |
| RRM1 | TCF7L2 |  |  |
| RUNX1 | TCL1A |  |  |
| RUNX1T1 | TET1 |  |  |
| SAMD9 | TET2 |  |  |
| SDHA | TFE3 |  |  |
| SDHB | TGFBR2 |  |  |
| SDHC | TGM7 |  |  |
| SDHD | TLR4 |  |  |
